# Mycobacterium avium complex: An unusual cause of hypercalcemia

**DOI:** 10.1016/j.idcr.2021.e01317

**Published:** 2021-10-25

**Authors:** Tulika Chatterjee, Yeshaswini Panathur Sreenivasa Reddy, Manasa Kandula

**Affiliations:** a Department of Internal Medicine, University of Illinois College of Medicine, Peoria, IL, USA; b Department of Gastroenterology, University of Illinois College of Medicine, Peoria, IL 61637, USA

**Keywords:** Hypercalcemia, Mycobacterium avium complex, Granulomatous disease, Immunocompetent

## Abstract

Mycobacterium avium-complex (MAC) is an infectious granulomatous disease which is associated with hypercalcemia especially in immunocompromised patients. We present an unusual case of MAC infection in an immunocompetent patient presenting as hypercalcemia.A 76-year-old immunocompetent male was admitted for hypercalcemia of 12.6 mg/dl found on outpatient evaluation for fatigue. PTH level was low 8 pmol/L, Vitamin D 25hydroxy was 29 ng/ml, 1,25 dihydroxy vitamin D (1,25(OH)2 vitamin D) levels was low at 11 pg/ml, PTH related peptide was 1.1 pmol/L. Hypercalcemia resolved with intravenous hydration and bisphosphonate administration. CT chest identified a nodule with central cavity in the right upper lobe. Pathology from percutaneous biopsy of thenodule demonstrated granulomatous inflammation. AFB culture came positive for MAC. Patient was treated with Azithromycin, Rifabutin and Ethambutol for twelve months.Granulomatous diseases like MAC cause hypercalcemia via activation of macrophages which express extrarenal 1- alpha -hydroxylase. It converts vitamin D to its active form 1,25(OH)2 vitamin D causing its excess, leading to hypercalcemia. Interestingly, in our patient calcium level was elevated with appropriately low PTH but 1,25(OH)2 vitamin D level was also low. There are few reported cases of hypercalcemia in granulomatous disease with normal levels of 1,25(OH)2 vitamin D levels, and our case is the first one to have MAC associated hypercalcemia with low 1,25(OH)2 vitamin D levels, suggesting an alternative mechanism for hypercalcemia in these patients

## Introduction

Mycobacterium avium-complex (MAC) infection is a granulomatous disease that can cause hypercalcemia and has been increasingly reported in the literature over the years, especially in immunocompromised patients [1]. The association between hypercalcemia and granulomatous disorders was first established in the year 1939 in sarcoidosis patients [2], [3]. Although the mechanism of hypercalcemia in granulomatous disease is thought to be excessive production of 1, 25 dihydroxycholecalciferol (1, 25 (OH) 2 vitamin D) by activated macrophages, this has not always been the case with MAC [1], [4]. Previous reports of MAC have described low or normal 1, 25(OH) 2 vitamin D levels suggestive of another mechanism contributing to hypercalcemia [4], [5]. We present a rare case of MAC presenting with isolated hypercalcemia and low 1, 25(OH) 2 vitamin D in an immunocompetent host.

## Case report

A 76-year-old male patient with a past medical history of chronic kidney disease stage 4, atrial fibrillation, and coronary artery disease was admitted for hypercalcemia of 12.6 mg/dl found on outpatient laboratory evaluation for fatigue. The levels remained elevated at 12.1 mg/dl on repeat testing at 48 h despite a dose of Pamidronate. This prompted the admission for further evaluation. He was on apixaban, allopurinol, amlodipine, donepezil, epoetin, esomeprazole, hydralazine, metoprolol, and simvastatin before admission. Of note, the patient used to be on calcitriol in the past but this was discontinued at least a month before admission and was unlikely to be related to the new hypercalcemia. He had complaints of chronic non-productive cough, fatigue, night sweats, and unintentional weight loss of 80 pounds within one year.

On physical examination, the patient was cachectic, afebrile, normotensive, and had dry mucosal membranes. Laboratory findings during admission were significant for a hemoglobin of 10.2 g/dl on the complete blood count. The metabolic panel was pertinent for elevated creatinine of 3.47 mg/dl increased from his baseline of 2.9 mg/dl, GFR of 17 ml/min, calcium level of 11.6 mg/dl, albumin of 2.7 mg/dl, and a mildly elevated alkaline phosphatase level of 73 U/L. Further workup revealed ionized calcium of 1.39 mmol/L, 25 hydroxy-vitamin D of 29 ng/ml, 1, 25 (OH) 2 vitamin D levels low at 11 pg/ml, PTH level low at 8 pmol/L, and PTH related peptide (PTHrP) unremarkable at 1.1 pmol/L.

**Fig. 1:**
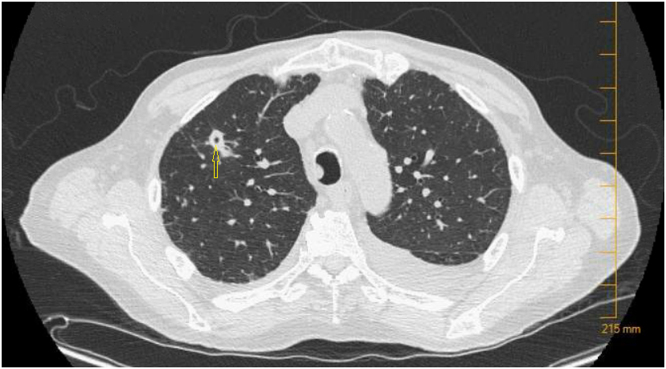
Computed tomography of the chest showed multiple, bilateral ill-defined nodular opacities. Yellow arrow pointing to a 1.3 × 0.9 cm nodule with central cavity in the right upper lobe.

Imaging modalities included a chest x-ray that showed new nodular opacities in the right upper and lower lobes along with another one in the left mid-lung. Subsequently, computed tomography of the chest (CT) showed multiple, bilateral ill-defined nodular opacities that were in a clustered configuration with some areas of branching. A 1.3 × 0.9 cm nodule with a central cavity in the right upper lobe and another 1.2 cm pleural-based nodule in the right posterior lobe were noted. There were multiple nonspecific sub-pleural ground-glass opacities and small bilateral pleural effusions (Fig. 1). The clustered configuration on the CT chest was suggestive of either a fungal or mycobacterial infection.

**Fig. 2:**
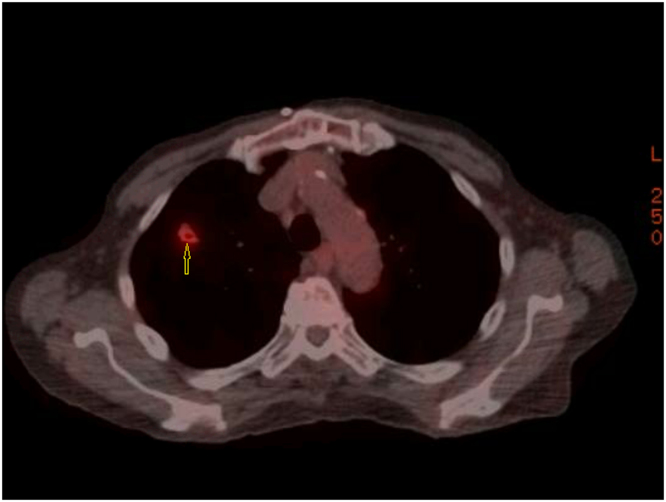
Positron emission tomography showing right upper lobe nodule with cavitation. Yellow arrow pointing to the cavity.

The patient underwent a CT-guided biopsy of the right upper lobe cavitating nodule. In the meantime, he also underwent a Positron Emission Tomography (PET) scan that showed multifocal hypermetabolic pulmonary nodules within both lungs, more on the right, and consistent with inflammation (Fig. 2). Gram stain of the biopsy tissue was negative, and the pathology report showed necrotizing and non-necrotizing granulomatous inflammation, along with occasional multinucleated giant cells, and Acid-Fast Bacillus (AFB) culture of the biopsy tissue was positive for MAC. The patient was initially treated with aggressive intravenous hydration which eventually led to the resolution of hypercalcemia. Once the diagnosis of MAC was confirmed, he was prescribed Azithromycin, Rifabutin, and Ethambutol thrice weekly for 12 months. He had steady improvement in all his symptoms with treatment of hypercalcemia and the underlying infection. Patient had normal calcium levels 1 year after the completion of treatment of MAC treatment and at that point we stopped following the patient.

## Discussion

Even though an uncommon clinical entity, MAC-associated hypercalcemia has been described extensively in HIV, lymphoma, and other immunocompromised patients but it is a rare phenomenon in immunocompetent patients [6], [7]. After a thorough literature review including PubMed, Medline, and Cochrane, we found only two other cases of MAC-induced hypercalcemia in immunocompetent patients [8], [9]. One of these cases of hypercalcemia was a patient with hot tub pneumonitis with elevated 1, 25(OH) 2 vitamin D [8], and the second case was a patient with MAC pneumonia with normal 1, 25(OH) 2 vitamin D [9]. To the best of our knowledge, the present case is the third reported case of MAC-associated hypercalcemia in an immunocompetent patient. Table 1 lists the cases of MAC-associated hypercalcemia in immunocompromised patients reported to date.

**Table 1:** Cases of MAC associated hypercalcemia in immunocompromised patients reported till date.

Author/year of publication	Immunocompromised status (Yes/No)	Diagnosis	Peak calcium level	PTH level	PTHrP level	Vitamin D 1, 25 dihydroxycholecalciferol level
Donato 2014 [9]	No	MAC hot-tub pneumonitis	11.9 mg/dl	15 pg/ml (Low normal)	14 pg/ml (Normal)	76 pg/dl (Elevated)
Parson 2017 [10]	No	MAC infection	12.8 mg/dl	8 pg/ml (Low)	1.8 pmol/L (Normal)	27 pg/ml (Normal)
Current case	No	MAC infection	12.6 mg/dl	8 pg/ml (Low)	1.1 pmol/L (Normal)	11 pg/ml (Low)

Hypercalcemia has been reported in granulomatous infectious diseases other than MAC, like tuberculosis, leprosy, fungal infections such as cryptococcosis, candidiasis, and coccidioidomycosis [8], [10]. The known mechanism of hypercalcemia in granulomatous diseases is an excessive conversion of 25-hydroxycholecalciferol to 1, 25(OH) 2 vitamin D [10]. Under normal physiological circumstances, 25- hydroxycholecalciferol is produced in the liver and is converted to 1, 25(OH) 2 vitamin D in kidneys by 1-alpha-hydroxylase, an enzyme produced by proximal renal tubular epithelial cells. 1, 25(OH) 2 vitamin D, in turn, facilitates calcium absorption by intestinal enterocytes and osteoclast-mediated bone resorption to raise calcium levels [11]. Renal 1-alpha hydroxylase is regulated by calcium, phosphorus, PTH, and 1, 25(OH) 2 vitamin D levels by feedback loop mechanisms [9]. In granulomatous diseases, the interstitial granulomas consist of activated macrophages that produce extra-renal 1-alpha hydroxylase, which leads to hypercalcemia (8). Besides macrophages, extrarenal 1-alpha hydroxylase can also be produced in the adrenal medulla, brain, pancreatic islet cells, intestinal cells, and placenta [10]. This extra-renal 1- alpha-hydroxylase is resistant to the negative feedback controls of calcium homeostasis and leads to persistent hypercalcemia [10].

Another interesting aspect of the current case is that our patient had hypercalcemia in the presence of low 1, 25(OH) 2 vitamin D. There are a few reported cases of hypercalcemia in patients with granulomatous disease where 1, 25(OH) 2 vitamin D levels are not elevated [5], [9]. Shrayyef et al. reported one case of sarcoidosis and another case of MAC in an HIV patient, both patients had hypercalcemia with a normal 1, 25(OH) 2 vitamin D [4]. The authors introduced the term “inappropriately normal” level of vitamin D despite low PTH, normal PTHrP, and phosphorus levels. These cases elucidate an alternate mechanism of hypercalcemia in granulomatous disease, other than through elevated 1, 25(OH) 2 vitamin D levels. Literature review showed authors have speculated other alternate mechanisms of hypercalcemia in granulomatous disease like elevated calcitonin levels and increased bone resorption with elevated alkaline phosphatase levels [4]. Under normal physiological conditions, 85% of 1, 25(OH) 2 vitamin D is bound to vitamin D binding proteins, 14.6% is bound to albumin and only 0.4% is in free form in blood [12]. But in 2010, Kalas et. al. published a case of extreme hypercalcemia associated with granulomatous myositis in the presence of normal 1,25 (OH)2D3 level and low PTH. He floated the concept of “relative excess” of1, 25(OH) 2 vitamin D, as he postulated that in cases of hypoalbuminemia, more free 1, 25(OH) 2 vitamin D becomes available to the tissues creating a discordance between serum and tissue levels of 1,25-dihydroxyvitamin D [5]. Few cases of hypercalcemia in granulomatous diseases with low 1, 25(OH) 2 vitamin D have been reported but not associated with MAC. Parker and Westphal reported cases of coccidioidomycosis associated with hypercalcemia with low 1, 25(OH) 2 vitamin D [13], [14]. A case of Mycobacterium marinum associated hypercalcemia with low 1, 25(OH) 2 vitamin D has been reported as well [15]. To the best of our knowledge, our case is the first reported case of MAC-associated hypercalcemia with low 1, 25(OH) 2 vitamin D levels.

Our patient had already received pamidronate outpatient and responded well to aggressive hydration which led to the resolution of hypercalcemia inpatient. In other case reports of MAC-associated hypercalcemia, patients responded to steroids [4], [8], [9]. Steroids potentially downregulate the production of activated vitamin D by the macrophages by decreasing the granulomatous inflammation and reduction in 1-alpha hydroxylase enzyme [1], [8]. Per some of the cases reported, bisphosphonates and calcitonin also have been used successfully to achieve normocalcemia [4]. Our patient did not need steroids for controlling hypercalcemia. Our patient received treatment for MAC which also led to the prevention of future episodes of hypercalcemia.

## Conclusion

Hypercalcemia is a common entity in both outpatient and inpatient settings, but an association with infectious causes such as MAC is very rarely considered. Although the exact mechanism for hypercalcemia in MAC with low 1, 25(OH) 2 vitamin D levels is still unknown, we present this case to increase awareness of this rare presentation. These patients should undergo appropriate diagnostic evaluation and be treated appropriately to prevent further complications.

## CRediT authorship contribution statement

**Tulika Chatterjee:** Writing – original draft, Contributed in writing the manuscript, Revised the manuscript. **Yeshaswini PS Reddy:** Contributed in writing the manuscript. **Manasa Kandula:** Ssupervised and edited the manuscript, Revised the manuscript.
